# Prazosin Can Prevent Glucocorticoid Mediated Capillary Rarefaction

**DOI:** 10.1371/journal.pone.0166899

**Published:** 2016-11-18

**Authors:** Erin R. Mandel, Emily C. Dunford, Anastassia Trifonova, Ghoncheh Abdifarkosh, Trevor Teich, Michael C. Riddell, Tara L. Haas

**Affiliations:** 1 School of Kinesiology and Health Science and Muscle Health Research Centre, York University, Toronto, Canada; 2 Department of Biology, York University, Toronto, Canada; "INSERM", FRANCE

## Abstract

Glucocorticoids (GC) elicit skeletal muscle capillary rarefaction, which can subsequently impair blood distribution and muscle function; however, the mechanisms have not been established. We hypothesized that CORT would inhibit endothelial cell survival signals but that treatment with the alpha-1 adrenergic receptor inhibitor prazosin, which leads to angiogenesis in skeletal muscle of healthy rats, would reverse these effects and induce angiogenesis within the skeletal muscle of corticosterone (CORT)-treated rats. Male Sprague Dawley rats were implanted subcutaneously with CORT pellets (400 mg/rat), with or without concurrent prazosin treatment (50mg/L in drinking water), for 1 or 2 weeks. Skeletal muscle capillary rarefaction, as indicated by a significant reduction in capillary-to-fiber ratio (C:F), occurred after 2 weeks of CORT treatment. Concurrent prazosin administration prevented this capillary rarefaction in CORT-treated animals but did not induce angiogenesis or arteriogenesis as was observed with prazosin treatment in control rats. CORT treatment reduced the mRNA level of Angiopoietin-1 (Ang-1), which was partially offset in the muscles of rats that received 2 weeks of co-treatment with prazosin. In 2W CORT animals, prazosin treatment elicited a significant increase in vascular endothelial growth factor-A (VEGF-A) mRNA and protein. Conversely prazosin did not rescue CORT-induced reductions in transforming growth factor beta-1 (TGFβ1 and matrix metalloproteinase-2 (MMP-2) mRNA. To determine if CORT impaired shear stress dependent signaling, cultured rat skeletal muscle endothelial cells were pre-treated with CORT (600nM) for 48 hours, then exposed to 15 dynes/cm^2^ shear stress or maintained with no flow. CORT blunted the shear stress-induced increase in pSer473 Akt, while pThr308 Akt, ERK1/2 and p38 phosphorylation and nitric oxide (NO) production were unaffected. This study demonstrates that GC-mediated capillary rarefaction is associated with a reduction in Ang-1 mRNA within the skeletal muscle microenvironment and that concurrent prazosin treatment effectively increases VEGF-A levels and prevents capillary loss.

## Introduction

Elevated circulating levels of glucocorticoids (GC) occurs in individuals with poorly controlled diabetes, metabolic syndrome or Cushing syndrome [1,2]. A prolonged pathophysiological increase in GC elicits a loss (rarefaction) of pre-existing capillaries within skeletal muscle [3]. Loss of skeletal muscle capillaries negatively impacts skeletal muscle function and reduces insulin sensitivity, thereby deteriorating overall cardiometabolic health [4–6]. The cellular mechanisms through which GC excess elicits the loss of skeletal muscle capillaries have not yet been established. Defining the cause of GC-induced skeletal muscle capillary rarefaction, and identifying means to prevent it, could improve skeletal muscle health and overall disease progression.

Capillary regression may occur due to inhibition of endothelial cell survival signals. The exposure of endothelial cells to the physical force of blood flow, shear stress, is a major physiological contributor to the promotion of endothelial cell survival and maintenance of a healthy vasculature [7,8]. Shear stress activates pro-survival factors such as Akt [9] and endothelial nitric oxide synthase (eNOS) [10]. This in turn will increase the production of nitric oxide (NO) [10], which exerts vasodilatory, anti-thrombotic and anti-inflammatory functions [11]. Vascular endothelial growth factor-A (VEGF-A) production is also increased by elevated shear stress [12] and exerts autocrine endothelial cell survival signaling [13].

Sustained elevation of blood flow is well characterized to induce vascular remodeling in the form of angiogenesis or arteriogenesis within healthy skeletal muscle [14,15]. Angiogenesis in response to elevated flow occurs through luminal division, or intussusception, rather than abluminal sprouting [7,16]. Both NO and VEGF are essential for the process of splitting angiogenesis [17,18]. Peripheral blood flow can be augmented experimentally via prazosin, an alpha-1 adrenergic receptor antagonist, which relieves the vasoconstrictor influences of the sympathetic nervous system on peripheral vascular beds. In healthy animals, prazosin increases femoral artery blood flow by approximately 60% [17] and the resultant increase in red blood cell velocity through the skeletal muscle capillary network is associated with an approximate threefold increase in shear stress [19]. In rodents, skeletal muscle capillary-to-fiber ratio (C:F) increases significantly after 7–14 days of prazosin treatment, indicative of an angiogenic response [7,20,21]. Arteriolar remodeling within the skeletal muscle microvascular network also occurs in response to elevated blood flow [22]. Arteriogenesis is likely to occur in response to prazosin treatment due to the findings of increased cellular proliferation at the arteriolar level after 4 days of prazosin treatment [23].

While the effects of alterations in shear stress on a healthy vasculature have been well established, little is known about the impact of chronic elevation in GC on shear stress-induced signaling pathways. To date, it is known that GC exposure does not induce apoptosis of cultured endothelial cells [3,24], but it was reported to promote microvascular endothelial cell death and capillary rarefaction *in vivo* [3,25]. Furthermore, GC impair sprouting angiogenesis by repression of endothelial cell proliferation, matrix proteolysis, sprouting and migration [3,26]. However, the influence of GC on the microvascular remodeling that typically is evoked by sustained increases in shear stress has not been investigated.

The first goal of this study was to identify alterations in the expression of vascular survival and angiogenesis-associated factors that could contribute to GC-induced capillary rarefaction. We hypothesized that GC would repress the expression of factors associated with endothelial cell survival. The second goal was to investigate the efficacy of prazosin-induced vascular remodeling in GC-treated animals. We hypothesized that prazosin treatment would reverse the influences of GC treatment on endothelial cell survival signaling, as well as induce angiogenesis within the skeletal muscle of GC-treated rats.

## Materials and Methods

All animal experiments were approved by York University Animal Care Committee (approval number 2013–5) and conducted in accordance with the Canadian Council for Animal Care Guidelines.

### Animal Protocol

Male Sprague Dawley rats (N = 48; initial weight 200-250g) were purchased from Charles River Laboratories (Montreal, QC, Canada). Rats were housed in the York University Vivarium in a 12-hour light-dark cycle. After 7 days acclimation, animals were assigned randomly to receive corticosterone or wax (control) pellets. Furthermore, animals were given either regular water or water containing prazosin hydrochloride (50 mg/L; P7791, Sigma Aldrich Canada) *ad libitum*. The four experimental groups were as follows: control-water, control-prazosin, CORT-water or CORT-prazosin. All animals were fed a standard rodent chow diet (14% protein, 54% carbohydrate, 32% fat; 3.0 calories/g) *ad libitum*. CORT (100mg) or wax pellets were made and on Day 0, four pellets were implanted subcutaneously in the mid-scapular region of each rat, under isoflurane anesthesia, as described previously [27]. Post pellet implantation, animals were provided with a subcutaneous injection of buprenorphine (0.02mg/kg) for analgesia. Subsequently, rodents recovered in individual cages and were given ampicillin (20 mg/kg) in their drinking water. On day 2, rats were continued with regular water or commenced with prazosin-treated water for 7 or 14 days. These groups will be referred to as 1W and 2W control or CORT- water or -prazosin.

### Plasma Corticosterone Analysis

Blood was collected via saphenous vein puncture at 8am on days 0, 7 and 14. Plasma CORT was measured by radio-immunoassay (07–120102, MP Biomedicals; Solon, OH) according to manufacturer’s instructions.

### Tissue Isolation

At the end point of each study, animals were anesthetized with inhaled isoflurane and skeletal muscles were removed. Muscles were subsequently weighed and snap frozen in liquid nitrogen or liquid nitrogen-cooled isopentane for further analysis, and animals were euthanized by exsanguination.

### Protein Extraction and Analysis via Western Blotting or ELISA

For western blots, protein was extracted from the *tibialis anterior* (TA) muscle or cultured endothelial cells in RIPA buffer including protease inhibitors and phosphoSTOP (Roche, Indianapolis IN). Muscle extracts were processed using a tissue lyser (MM400, Retsch GmbH, Haan, Germany). Subsequently, protein extracts were quantified by bicinchoic acid assay (Pierce, Fisher Thermoscientific). 20–40 μg of total proteins were separated through 8% or 10% SDS-polyacrylamide gels under reducing conditions. Proteins were transferred to PVDF membrane (Immobilon-P, Millipore) using wet transfer (Biorad trans-blot) and membranes were blocked for 1hr with 5% milk in TBS- 0.05% Tween (TTBS). Membranes were incubated overnight at 4°C with antibodies for phospho-Akt Ser473, Thr308 (#4058 & 9275, Cell Signaling), phospho-ERK1/2 (# 9101, Cell Signaling), phospho-p38 (#9211, Cell Signaling) or TSP-1 (#MA5-13398, Thermo Scientific) in 5% BSA -TTBS. Secondary antibodies (rabbit IgG-HRP) were detected by enhanced chemiluminescence (Pierce WestPico, Fisher Thermoscientific). Membranes were stripped and re-probed for total AKT (# 9272, Cell Signaling), total p38 (#9272, Cell Signaling) anti-β-actin (1:5000, Santa Cruz Biotechnology, Santa Cruz CA) or α/β tubulin (#2148, Cell Signaling) for normalization. Blots were quantified and analyzed using Carestream software.

For the VEGF-A ELISA, *gastrocnemius* muscle was homogenized in PBS using a tissue lyser. On the same day, 100μg protein per sample was assayed by ELISA (R&D systems, #RRV00), as per the manufacturer’s instructions.

### RNA extraction and Real Time qPCR

RNA was isolated from TA muscle using the Qiagen RNeasy Fibrous Tissue Mini Kit (74704, Qiagen, Toronto, ON Canada) as per the manufacturer’s instructions. RNA was reverse transcribed using MMLV reverse transcriptase (New England Biolabs, Whitby ON Canada). cDNA were analyzed by Taqman qPCR using qPCR mastermix (#4444963; Invitrogen Canada) and Taqman probes for rat HPRT1 (Rn01527840_m1), VEGF-A (Rn00582935_m1), TSP-1 (Rn01513693_m1), Ang1 (Rn01504818_m1 or Mm00456503_m1), Ang 2 (Mm00545822_m1), MMP-2 (Mm00439498_m1), TIMP1 (Rn00587558_m1) and TGFβ1 (Rn99999016_m1) using the ABI 7500 Fast PCR system (Invitrogen Canada). For each sample, the comparative Ct method (2^-ΔCt^) was used to determine relative mRNA expression of target genes relative to the housekeeping gene HPRT1.

### Muscle Histology and cell staining

Cryosections of TA muscle (10μm) were fixed with 3.7% paraformaldehyde and stained with fluorescein isothiocyanate-conjugated *Griffonia simplicifolia* isolectin B4 (1:100; Vector Laboratories, Burlington ON, Canada) and anti-smooth muscle actin-Cy3 (1:300; C6198, Sigma Aldrich, Oakville ON, Canada). Sections were viewed using a Zeiss M200 inverted microscope with 20x objective and images were captured using a cooled CCD camera using Metamorph imaging software. Capillary-to-fiber (C:F) counts were averaged from 3–4 independent fields of view per rat by a blinded observer. The density and diameters of vessels that stained positive for smooth muscle actin (SMA^+^) also were calculated from the same images. The average diameter of SMA^+^ vessels (discounting large thin-walled venular structures) was calculated from 3–4 independent fields of view per rat, as an indicator of outward remodeling of arterioles. The density of SMA^+^ vessels under 20μm was assessed, as this size of arterioles is most likely to increase in number if capillaries undergo conversion to arterioles [28].

### Cell culture and shear stress stimulation

Skeletal muscle microvascular endothelial cells were isolated from the *extensor digitorum longus* (EDL) muscle of male Sprague Dawley rats as described previously [29] and cultured with Dulbecco’s Modified Eagle Medium (DMEM, Invitrogen) supplemented with 10% heat denatured FBS, 1mM sodium pyruvate, 1mM Glutamax (Invitrogen), 50 units penicillin, 0.5mg/ml streptomycin and 1.25μg/ml fungizone. Endothelial cells were used for experiments between passages 4 to 7. Endothelial cells were plated on 35mm dishes or gelatin-coated glass cover slips and pre-treated with 600 nM CORT for 48 hours, a dose that previously was shown to inhibit angiogenic behavior [3,26]. Shear stress experiments were conducted as previously described [30]. Briefly, cells were subjected to 15 dynes/cm^2^ shear stress for 2 hours using a parallel plate flow system (Bioptechs), to provide a stimulus comparable to reported capillary shear stress within skeletal muscle of prazosin-treated rats [19].

C2C12 murine myoblasts were differentiated into myotubes by culturing in basal DMEM supplemented with 5% horse serum. Myotubes were treated with 600 nM CORT for 48 hours prior to cell lysis for RNA extraction.

### Statistical Analysis

Results were expressed as mean ± SEM and analyzed by one- or two-way ANOVA with subsequent Bonferroni post hoc tests as appropriate (Prism4; Graphpad software Inc; La Jolla, CA, USA). *P* < 0.05 was considered statistically significant.

## Results

### CORT-induced reduction in skeletal muscle capillarization is abrogated with prazosin treatment

**Fig 1:**
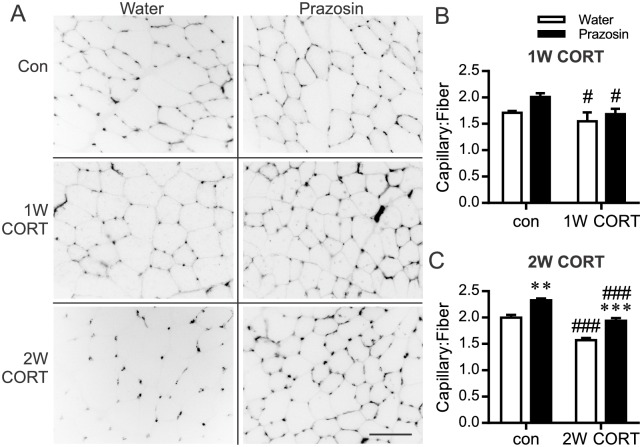
Corticosterone-induced capillary rarefaction is abrogated by continuous prazosin treatment. TA muscle were sectioned and stained for capillaries using fluorescein*-Griffonia Simplicifolia* isolectin and Cy3-anti-α smooth muscle actin. (A) Representative images of isolectin staining after 1W and 2W of CORT-treatment. Inverted grey scale images of isolectin staining are displayed to enhance visualization of individual muscle fibers. Scale bar represents 100 μm. (B) C:F at the 1W and (C) 2W time points was calculated from the average of 5 non-overlapping fields of view, ^#^*P*<0.05 1W CORT vs. corresponding control group; ***, ^###^*P*<0.001 2W CORT vs. corresponding water or control group respectively, n = 6–9.

Plasma CORT was significantly elevated above control after both 1W and 2W of exogenous CORT pellet treatment, and was not influenced by prazosin administration (Table 1). Both 1W and 2W CORT significantly reduced skeletal muscle C:F as compared to time-matched control animals (Fig 1A–1C). Two weeks of prazosin treatment elicited a significant increase in C:F in control animals (Fig 1A and 1C). Concurrent prazosin treatment prevented CORT-dependent capillary rarefaction (Fig 1A and 1C). Nonetheless, C:F remained significantly lower in muscles of CORT-prazosin compared to control-prazosin rats.

**Table 1:** Plasma CORT concentration.

	**1 Week**			
Plasma corticosterone(ng/ml)	**Control-Water(n = 5)**	**Control-Prazosin(n = 5)**	**CORT-Water(n = 5)**	**CORT-Prazosin(n = 5)**
	17.54±1.4	18.94±5.5	583.1±39.8^#^	485.5±72.3^#^
	**2 Weeks**			
Plasma corticosterone(ng/ml)	**Control-Water(n = 5)**	**Control-Prazosin(n = 5)**	**CORT-Water(n = 9)**	**CORT-Prazosin(n = 6)**
	16.21± 6.5	9.6 ±0.6	296.3±34.4^#^	284.7±34.2^#^

Values are expressed as mean ± standard error

^#^ *P*<0.05 compared to respective control group, Bonferroni post hoc analysis

### Factors associated with CORT-mediated capillary rarefaction

**Fig 2:**
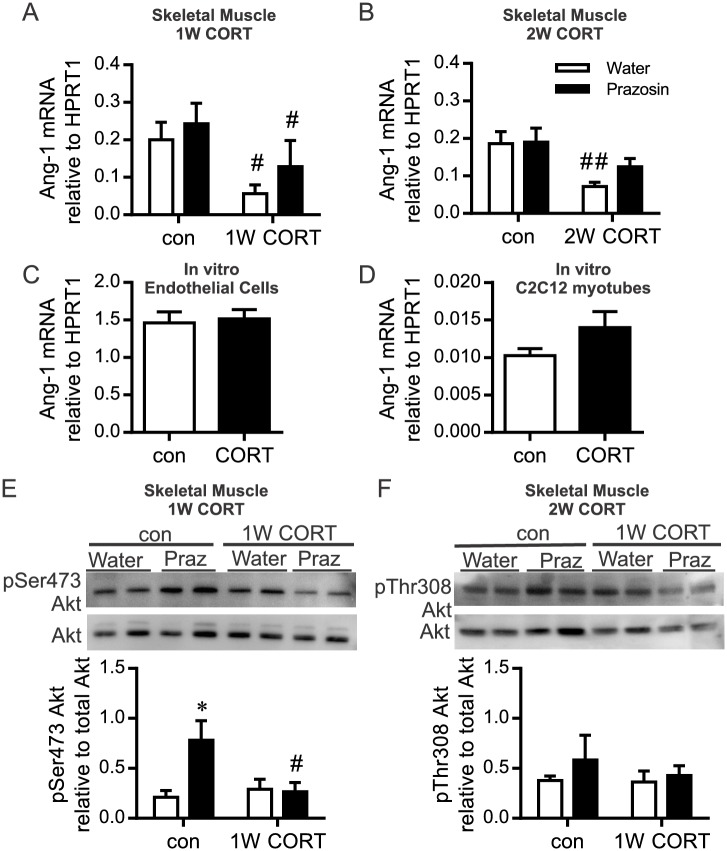
CORT influence on Ang-1 mRNA and Akt phosphorylation. RNA was isolated from the TA muscle after 1W or 2W of CORT treatment with or without concurrent prazosin or from cultured endothelial or C2C12 cell extracts after 48 hours of CORT-treatment. (A,B) Ang-1 mRNA, assessed by Taqman qPCR and represented as 2^-ΔCt^ relative to the housekeeping gene HPRT1, was significantly reduced with CORT treatment (^#^*P* = 0.02; main effect of 1W CORT; ^##^*P*<0.05 2W CORT vs. control water, n = 4–8). Ang-1 mRNA was unaltered within cultured endothelial cells (C) or in differentiated C2C12 myotubes (D) after 48 hours of CORT-treatment (*P* = 0.85 and *P* = 0.18 respectively, n = 5–6). Protein was extracted from the TA muscle of control or 1W CORT treated rats. Changes in phosphorylated Ser473-Akt (E) and Thr308-Akt (F) were assessed using Western blot and normalized to levels of total Akt. (E) A significant interaction between prazosin and CORT treatment was detected for pSer473-Akt (^#^*P*<0.05). Post hoc analysis indicated a significant difference between water and prazosin in the control-treated animals (*P<0.05). (F) No significant changes were detected in pThr308-Akt (n = 4–8).

We assessed factors associated with endothelial cell survival and angiogenesis that might be associated with CORT-induced capillary rarefaction. Angiopoietin-1 (Ang-1) mRNA was decreased significantly after both 1 and 2 weeks of CORT treatment (Fig 2A and 2B). Ang-1 mRNA level in 2W prazosin-treated CORT rats was no longer different from control levels (Fig 2B). However, 48 hours of elevated CORT was insufficient to alter the level of Ang-1 mRNA either in cultured endothelial cells or differentiated C2C12 myotubes (Fig 2C and 2D). Ang-2 mRNA was undetectable in whole muscle lysates.

Akt is a downstream effector of Ang-1/Tie2 signaling [31]. 1W CORT did not significantly alter basal phosphorylation of Akt at Ser473 or Thr308 (Fig 2E and 2F). Of note, the prazosin-induced increase in pAkt Ser473 seen in wax animals was absent in CORT-treated animals (Fig 2E), while no significant differences were observed in pAKT Thr308 (Fig 2F). No changes in Akt phosphorylation (Ser473 or Thr308) were detectable in the 2W treated muscles (data not shown).

**Fig 3:**
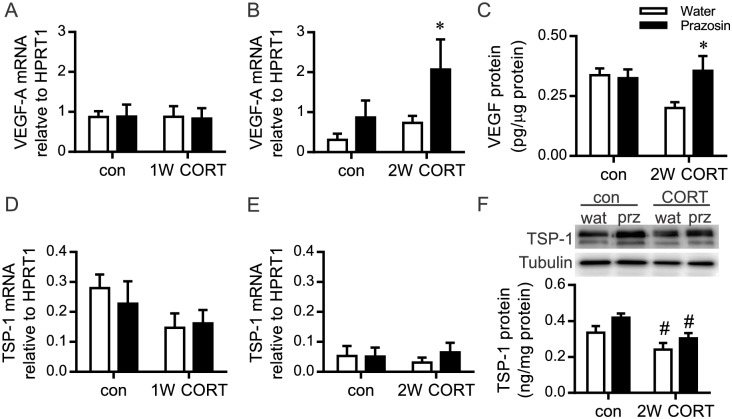
Alterations to VEGF-A and TSP-1 with elevated CORT and concomitant prazosin treatment. RNA or protein was isolated from the TA muscle after 1W or 2W of CORT with or without concurrent prazosin treatment. Taqman qPCR was used to assess the mRNA levels of VEGF-A (A,B) and TSP-1 (D,E), while VEGF-A protein was assessed by ELISA (C) and TSP-1 protein by Western blot (F). (A) VEGF-A mRNA was not altered in response to 1W CORT and/or prazosin. (B) VEGF-A mRNA was not affected by 2W CORT (*P* = 0.08), while a significant prazosin effect was detected within the 2W CORT-prazosin group (*P<0.05). (C) At the 2W time point, VEGF-A protein displayed a tendency for a CORT effect (*P* = 0.08), and was significantly increased in the CORT-prazosin cohort compared to water CORT animals (*P<0.05). (D, E) TSP-1 mRNA was not altered by 1W or 2W CORT and/or prazosin. (F) TSP-1 protein level was significantly reduced with 2W CORT treatment (^#^*P*<0.05), and there was a trend (*P* = 0.09) for an increase in TSP-1 after 2W of prazosin treatment within control animals (n = 4–9).

Neither 1W or 2W CORT treatment significantly reduced VEGF-A mRNA (Fig 3A and 3B). Conversely, VEGF-A mRNA levels were increased significantly in the 2W CORT-prazosin compared with CORT-water muscles (Fig 3B). Similarly, VEGF-A protein was significantly elevated at the 2W time point in CORT-prazosin compared to CORT-water treated rats (*P*<0.05, Fig 3C).

The mRNA level of the angiostatic factor thrombospondin 1 (TSP-1) was not altered by 1W or 2W of CORT-treatment or by prazosin treatment (Fig 3D and 3E). 2W of CORT treatment elicited a small but significant reduction in TSP-1 protein level, with no effect of concurrent prazosin treatment (Fig 3F).

### Prazosin-induced arteriolar remodeling is impaired in skeletal muscle of CORT-treated rats

**Fig 4:**
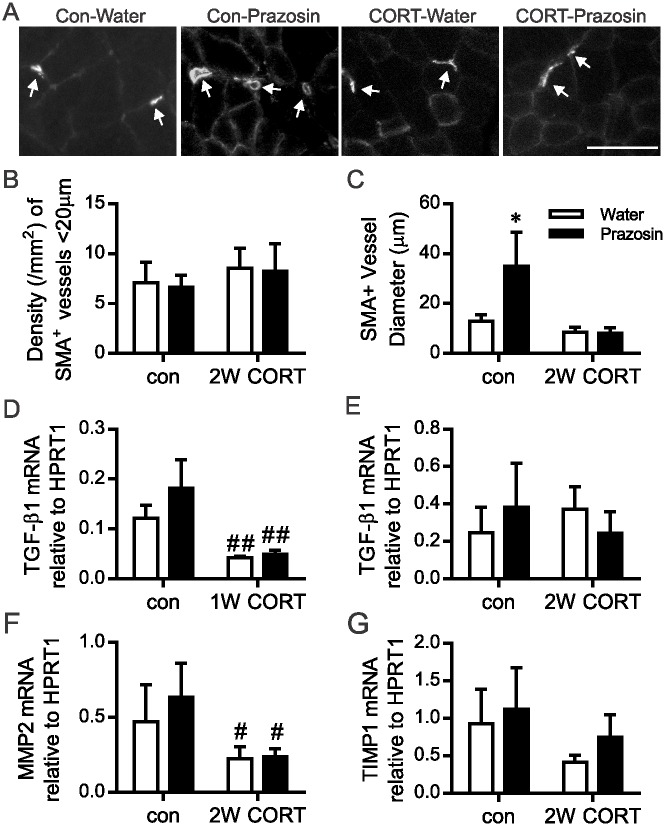
CORT-induced inhibition of flow induced arteriolar remodeling is not reversed with prazosin treatment. Histological analysis of SMA^+^ vessels within the TA muscle at the 2W time point was calculated from 5 non-overlapping fields of view per rat. (A) Representative images of SMA^+^ staining at the 2W timepoint. Grey scale images are displayed to enhance visualization of individual muscle fibers. Arrows point to SMA^+^ vessels in each condition. Scale bar represents 100 μm. (B) Average SMA^+^ density (per mm^2^) was unaltered by CORT and/or prazosin treatment. (C) 2W of concurrent prazosin treatment caused a significant increase in average SMA^+^ vessel diameter in control, but not in CORT-treated, animals (*P<0.05 vs. corresponding water group; n = 5–9). The mRNA levels of key arteriogenic factors within the TA muscle was assessed by Taqman qPCR and expressed as 2^-ΔCt^ relative to HPRT1. (D, E) TGFβ1 mRNA was repressed after 1W CORT (^#^*P*<0.05), which was not alleviated by concurrent prazosin treatment. Neither CORT nor prazosin influenced TGFβ1 mRNA at the 2W time-point. (F) MMP-2 mRNA level was repressed by 2W CORT (^#^*P*<0.05). (G) TIMP1 mRNA was unaltered by 2W CORT or prazosin (n = 5–9).

To explore the potential impact of CORT and prazosin treatment on arteriolar remodeling, we assessed the density and diameter of SMA^+^ vessels (Fig 4A–4C). The density of small SMA^+^ blood vessels was unaltered by 2 weeks of prazosin or CORT treatment (Fig 4B). In contrast, an increase in SMA^+^ vessel diameter was detectable following prazosin-treatment, but only within control animals (Fig 4C). Shear stress-stimulated outward remodeling of blood vessels can be promoted by TGFβ1 signaling [32] and MMP activity [33]. 1W CORT treatment significantly blunted the level of TGFβ1 mRNA, but this influence was not detected after 2 weeks of treatment (Fig 4D and 4E). We previously reported that CORT treatment repressed MMP-2 expression in cultured endothelial cells [3]. Consistent with the lack of arteriolar remodeling, we observed that MMP-2 mRNA was reduced significantly within the skeletal muscle of CORT treated rats and was not affected by concomitant prazosin treatment (Fig 4F). TIMP1 mRNA, an endogenous inhibitor of MMP proteolytic activity, was not altered by CORT or prazosin in 2W treated animals (Fig 4G).

### CORT-dependent regulation of pro-survival and angiogenic factors in endothelial cells

**Fig 5:**
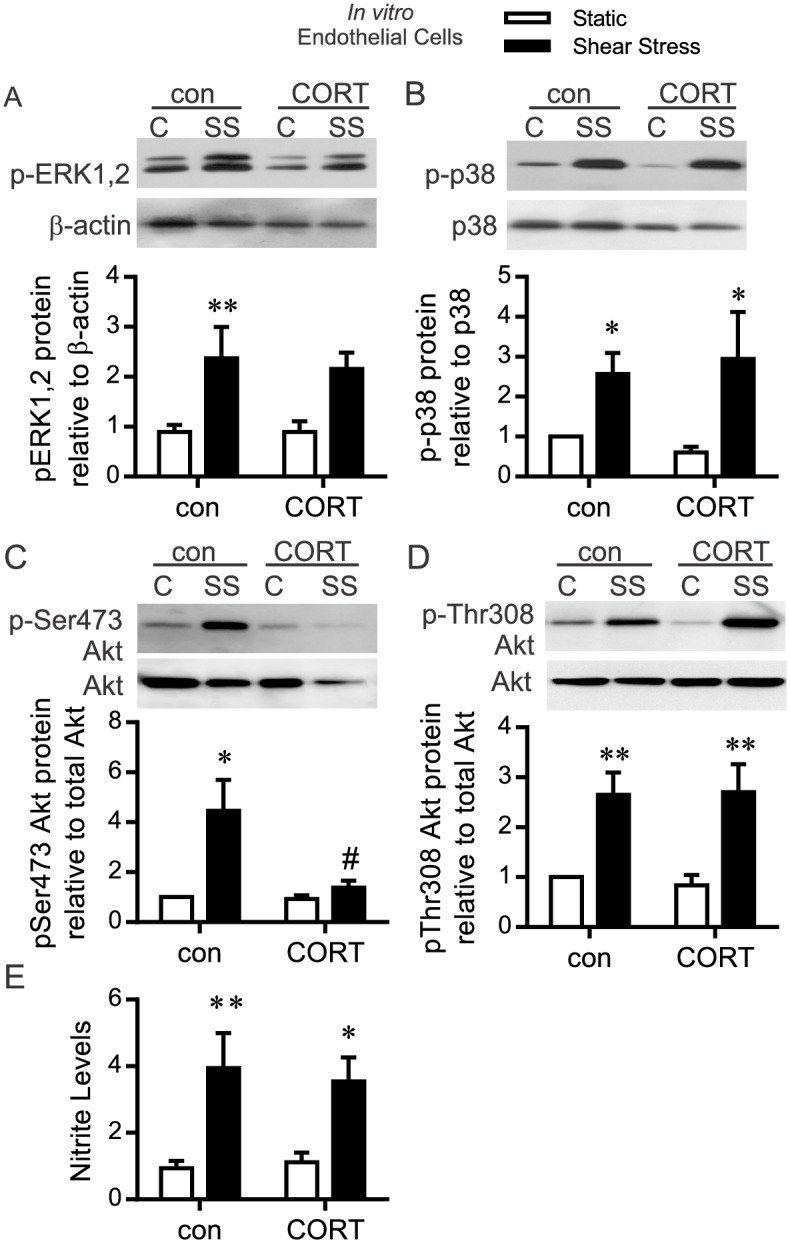
Influence of corticosterone on endothelial specific shear stress responsiveness. Cultured rat skeletal muscle endothelial cells were pre-treated with corticosterone (600 nM) for 48 hours prior to shear stress (15dynes/cm^2^), or were maintained in static conditions (C), for 2 hours. (A-D): Whole cell lysates were analyzed by Western blotting. (A) Phospho-ERK1,2 protein level relative to β-actin. (B) Phospho-p38 protein level relative to total p38. (C) Phospho-Ser473 Akt protein level relative to total Akt. (D) Phospho-Thr308 Akt protein level relative to total Akt. Two-way ANOVA indicated a significant shear effect for all kinases (pERK1,2: *P* = 0.007; pp38: *P* = 0.02; pSerAkt: *P* = 0.02; pThrAkt: *P* = 0.0003, n = 3–4). *,** *P*<0.05, *P*<0.01 compared to respective static control, post hoc analysis. A significant interaction between shear stress and CORT was detected only for pAktSer473 (^#^*P* = 0.05). (E) Nitric oxide level was assessed indirectly by Griess assay. A main effect of shear stress was detected (*P* = 0.0006; n = 6). (*,**P<0.05 and *P*<0.01, relative to static controls as assessed via post hoc analysis).

Due to the impairment of normal blood flow remodeling responses in CORT-treated animals, we used cultured endothelial cells to more specifically assess the influence of CORT on endothelial cell shear stress-dependent signaling. Shear stress stimulates endothelial cell phosphorylation of ERK1/2 and p38 MAPK [34] and Akt [9]. CORT did not attenuate the shear stress-stimulated phosphorylation of ERK1/2 or p38 MAPK (Fig 5A and 5B). Interestingly, CORT treatment abolished the shear stress mediated phosphorylation of Akt at Ser473 while phosphorylation at Thr308 was unaffected (Fig 5C and 5D). The shear stress-induced increase in nitrite levels, a measure of NO production, was not altered by CORT (Fig 5E).

## Discussion

We report for the first time that CORT-mediated skeletal muscle capillary rarefaction was associated with lower levels of Ang-1, but not VEGF-A mRNA, and that capillary loss was prevented with 2W of concurrent prazosin treatment. While prazosin was able to prevent CORT-induced capillary rarefaction, the normal angiogenic and arteriogenic responses to prazosin were absent in CORT-treated animals. Elevated CORT resulted in a decrease in Ang-1 expression, which may correspond with the observed rarefaction of capillaries within skeletal muscle. Conversely, the prazosin-induced increase in VEGF-A mRNA and protein in CORT-treated animals may contribute to the maintenance of capillary number observed with prazosin treatment. Shear stress responses were largely unaffected in cultured endothelial cells treated with CORT, with the exception of Ser473-Akt phosphorylation, suggesting that the failure to undergo angiogenesis and arteriogenesis is not a consequence of impaired shear stress responsiveness. Taken together, our findings indicate that high levels of circulating CORT negatively impact the skeletal muscle microenvironment, thereby promoting capillary rarefaction and impairing vascular remodeling responses. These data further suggest that the sustained increase in skeletal muscle blood flow via prazosin treatment can mitigate CORT-induced capillary rarefaction.

Alterations in skeletal muscle capillary number correlate positively with insulin sensitivity [35–37], as capillary content establishes the efficient delivery of insulin to skeletal myocytes[38]. This fundamental relationship illustrates the physiological importance of assessing the causes of, and defining the means to prevent, CORT-induced capillary rarefaction. The loss of capillaries in response to CORT treatment suggests a reduction in pro-survival signaling. We detected a decrease in Ang-1 after both 1W and 2W of CORT treatment, which paralleled CORT-mediated capillary rarefaction. Ang-1 maintains a quiescent endothelium, as well as endothelial cell structural integrity and survival through its activation of the Tie2 receptor [39–41], while Ang-2 is thought to compete for Tie2 binding, thus disrupting Ang-1 signaling [42]. However, in the current study we report that Ang-1 expression was not altered by 48 hr CORT treatment in cultured endothelial cells or myotubes, which suggests that CORT does not exert direct transcriptional repression of Ang-1. We were unable to detect Ang-2 mRNA levels in the whole muscle; however, we cannot discount that the reduction in Ang-1 detected with CORT treatment facilitates a greater functional role for Ang-2. Several of the cellular effects of Ang-1 are mediated through activation of the PI3K/Akt signaling pathway [43]. No change in the basal level of Akt phosphorylation (Ser473 or Thr308) was noted with CORT treatment. Thus, it is unlikely that a reduction in basal Akt activity underlies the CORT-induced capillary rarefaction, despite the established role of Akt in cell survival, proliferation and cell cycle progression [43–45].

Endothelial cell derived VEGF-A has been shown to act as an important autocrine pro-survival signal [13] and the knock-down of skeletal myocyte-derived VEGF-A causes substantially reduced muscle capillarization [46]. Nonetheless, we did not detect a reduction in skeletal muscle VEGF-A mRNA or protein after 1 or 2 weeks of CORT treatment, although we previously reported that 48 hr GC treatment reduced VEGF-A mRNA in cultured endothelial cells [3]. Furthermore, the potent anti-angiogenic factor TSP-1 [47,48] was not significantly increased by CORT treatment at any of the time points examined. A previous study reported a transient, but not sustained, GC- mediated increase in TSP-1 expression [49], and such an effect would have not been captured in the time frame of our study. Thus, our data indicate that the repression of Ang-1 expression, rather than alterations in VEGF-A or TSP-1, is the most plausible contributor to CORT-mediated capillary rarefaction. However, the role that Ang-1 might play in GC-mediated capillary rarefaction remains to be established.

In our study, prazosin treatment was used as a tool to examine the efficacy of flow dependent remodeling within the skeletal muscle microvasculature in CORT-treated rats. Notably, 2-weeks of concurrent prazosin treatment effectively prevented CORT-induced capillary rarefaction. A partial recovery of Ang-1 mRNA level was detected at this time point. Nonetheless, the alteration in Ang-1 mRNA with prazosin treatment was modest and by itself, may not explain the lack of capillary rarefaction. The concomitant restoration of VEGF-A protein level in 2W CORT-prazosin treated animals may also contribute significantly to the prevention of capillary rarefaction.

While the expected prazosin-induced angiogenic and arteriogenic responses occurred within the skeletal muscle of control animals, they were absent in prazosin-treated CORT animals. Shear stress-induced angiogenesis is mediated, in part, by an increase in pro-angiogenic signaling involving VEGF-A [19,50] and a down-regulation of anti-angiogenic factors such as TSP-1 [51]. While we did not detect an increase in VEGF-A mRNA in control/prazosin treated animals, it is likely this is due to the time point analyzed, since VEGF-A mRNA was increased at 2 days of prazosin treatment in healthy rats [12]. Interestingly, VEGF-A mRNA increased after 2W of prazosin treatment within the CORT group, which could be an indication that the angiogenic response to prazosin was delayed, and might have been detected if prazosin treatment were continued beyond 2-weeks.

Prazosin induces arteriolar remodeling analogous to the outward remodeling that occurs in larger arteries exposed to elevated shear stress [52–54]. The absence of prazosin-induced arteriogenesis in CORT-animals is corroborated by previous work which found that dexamethasone treatment impaired arteriogenesis in a mouse model of hind limb ischemia [55]. Arterial remodeling is regulated, in part, by TGFβ1 and MMP signaling [32,33]. In the current study, *in vivo* CORT treatment transiently reduced the mRNA levels of TGFβ1 and prazosin treatment did not fully rescue the expression of TGFβ1. A more pronounced and sustained reduction in MMP-2 expression was detected in CORT-treated animals, as has been reported in previous *in vitro* studies [3,56], and this repression was not relieved by prazosin treatment. We did not detect changes in TIMP1 mRNA with CORT treatment, although GC were reported to increase TIMP1 mRNA in cultured cerebral endothelial cells [57]. Taken together, the reductions in TGFβ1 and MMP-2 portray a scenario in which CORT alters the microvascular environment and represses flow-induced outward arteriolar remodeling by reducing the production of factors that can promote smooth muscle cell proliferation and extracellular matrix proteolysis.

The absence of flow-remodeling responses to prazosin in CORT-treated animals could be attributed to impaired shear stress-activation of angiogenic signals. *In vitro* endothelial cell culture experiments support the conclusion that CORT-treated endothelial cells generally retain shear stress responsiveness of major cell signal pathways, such as pERK1/2 and p38MAPK. Interestingly, CORT did blunt pSer473 Akt phosphorylation in response to shear stress, both *in vitro* and *in vivo*. Evidence exists that phosphorylation at Thr308, and not Ser473, is required for Akt activation and the promotion of several downstream signaling pathways [58], thus the functional consequence of reduced pSer473 Akt within the context of our study is uncertain. Although we did not detect an alteration in shear stress-induced NO production, a known downstream effector of Akt signaling [10], we cannot exclude a possible influence on other Akt effectors that may have an impact on capillary maintenance. It also is possible that CORT exerts influences through shear stress signaling pathways that were not assessed in the current study. Taken together these findings suggest that shear stress responsiveness is maintained although it is slightly blunted due to CORT pre-treatment.

A limitation of the current study is that it did not include an assessment of muscle blood flow. The lack of angiogenesis and arteriolar remodeling within CORT-treated animals may reflect a failure to increase blood flow to the threshold level required for adaptive microvascular remodeling. CORT has been previously shown to elicit a reduction in blood flow within the temporal lobe [59]. Thus, it is possible that prazosin treatment restored muscle blood flow within CORT-treated animals to normal baseline levels. As such, this level of shear stress was sufficient to prevent CORT-induced capillary rarefaction but not to induce further capillary growth. The majority of mRNA/protein within the skeletal muscle homogenate is derived from skeletal myocytes and endothelial cells, based on their quantitative representation within muscle. However, it is recognized that the skeletal muscle microenvironment includes not only skeletal myocytes and vascular cells, but also nerve fibers and interstitial cells that could contribute to the observed effects of CORT and prazosin on the vasculature. Our study design did not enable us to determine the involvement of specific cell types to the overall effects of CORT. Finally, a larger study group may have enabled the elucidation of additional significant alterations in key angiogenic regulators, given the variability detected between animals.

The current study demonstrates that CORT treatment evokes alterations within the skeletal muscle microenvironment that contributes to the loss of skeletal muscle capillaries. Our data indicate that a sustained reduction in Ang-1 expression may be related to the capillary rarefaction that is caused by GC-excess. Prazosin prevented CORT-induced capillary rarefaction, which may be a consequence of an increase in VEGF-A mRNA and protein. However, CORT treatment blocked the typical prazosin-induced angiogenic and arteriogenic responses. Taken together, these findings suggest that prazosin may offset the deleterious effect of elevated levels of GC on skeletal muscle capillary content but that it has limited capacity to increase capillary number in the continued presence of GC.
